# Comparative Incidence of Conformational, Neurodegenerative Disorders

**DOI:** 10.1371/journal.pone.0137342

**Published:** 2015-09-03

**Authors:** Jesús de Pedro-Cuesta, Alberto Rábano, Pablo Martínez-Martín, María Ruiz-Tovar, Enrique Alcalde-Cabero, Javier Almazán-Isla, Fuencisla Avellanal, Miguel Calero

**Affiliations:** 1 Department of Applied Epidemiology, CNE, National Institute of Health Carlos III, Madrid, Spain; 2 Consortium for Biomedical Research in Neurodegenerative Diseases (CIBERNED), National Institute of Health Carlos III, Madrid, Spain; 3 Alzheimer's Disease Centre, Reina Sofia Foundation, Madrid, Spain; 4 CIBERNED, National Institute of Health Carlos III, Majadahonda, Spain; FDA, UNITED STATES

## Abstract

**Background:**

The purpose of this study was to identify incidence and survival patterns in conformational neurodegenerative disorders (CNDDs).

**Methods:**

We identified 2563 reports on the incidence of eight conditions representing sporadic, acquired and genetic, protein-associated, i.e., conformational, NDD groups and age-related macular degeneration (AMD). We selected 245 papers for full-text examination and application of quality criteria. Additionally, data-collection was completed with detailed information from British, Swedish, and Spanish registries on Creutzfeldt-Jakob disease (CJD) forms, amyotrophic lateral sclerosis (ALS), and sporadic rapidly progressing neurodegenerative dementia (sRPNDd). For each condition, age-specific incidence curves, age-adjusted figures, and reported or calculated median survival were plotted and examined.

**Findings:**

Based on 51 valid reported and seven new incidence data sets, nine out of eleven conditions shared specific features. Age-adjusted incidence per million person-years increased from ≤1.5 for sRPNDd, different CJD forms and Huntington's disease (HD), to 1589 and 2589 for AMD and Alzheimer's disease (AD) respectively. Age-specific profiles varied from (a) symmetrical, inverted V-shaped curves for low incidences to (b) those increasing with age for late-life sporadic CNDDs and for sRPNDd, with (c) a suggested, intermediate, non-symmetrical inverted V-shape for fronto-temporal dementia and Parkinson's disease. Frequently, peak age-specific incidences from 20–24 to ≥90 years increased with age at onset and survival. Distinct patterns were seen: for HD, with a low incidence, levelling off at middle age, and long median survival, 20 years; and for sRPNDd which displayed the lowest incidence, increasing with age, and a short median disease duration.

**Interpretation:**

These results call for a unified population view of NDDs, with an age-at-onset-related pattern for acquired and sporadic CNDDs. The pattern linking age at onset to incidence magnitude and survival might be explained by differential pathophysiological mechanisms associated with specific misfolded protein deposits.

## Introduction

Within the wide field of neurodegenerative disorders (NDDs), encompassing both highly frequent and rare ailments, the large aetiological NDD subgroup known as sporadic NDDs (sNDDs) constitutes a major health problem in the industrial world. Expectations of the possibility of NDD prevention at the present time are poor. The term *sporadic*, equivalent to *idiopathic*, essentially denotes the absence of a known cause, as opposed to the *genetic* and *acquired* categories, which complete the aetiological spectrum and are well established only in prion-related NDDs. The sNDD group embraces a variety of disorders, such as the sporadic forms of Alzheimer's disease (AD), essential tremor (ET), idiopathic Parkinson's disease (PD), Lewy body disease (LBD), fronto-temporal dementia (FTD) and amyotrophic lateral sclerosis (ALS), as well as a few rare, rapidly progressive disorders, among which sporadic Creutzfeldt-Jakob disease (sCJD) and non-prion sporadic rapid-progressive neurodegenerative dementia (sRPNDd) predominate [1,2]. Since the late 1990s, the advent of commercially available antibodies has paved the way for new protein-identification techniques. Such advances have led to a considerable expansion of knowledge of protein misfolding, aggregation and deposit, and an ensuing improvement in our understanding of the molecular pathophysiology of neurodegeneration and the interpretation of epidemiological NDD data.

Authors have acknowledged that *templating*, the mechanism generating protein misfolding and cell-to-cell transfer of the pathogenic protein conformation, must be assumed to constitute a general phenomenon relevant to the epidemiology of misfolded protein-associated or so-called conformational NDDs (CNDDs) [3]. Little attention has, however, been paid to the validity of incidence measurements, and in particular, to the epidemiological view across entities from the standpoint of their pathognomonic protein signatures and morphological features. For instance, the low positive predictive value of PD diagnosis, i.e., 76% [4], in the 1990s, when many PD screening surveys were conducted, and the presence of neuritic Aβ plaques or tau deposits in considerable proportions of non-demented persons [5,6] seriously undermine the validity of numerators and denominators of clinical PD and AD incidence counts respectively. Bearing such limitations in mind, we believe that a comparative reappraisal of the incidence of CNDDs is called for, particularly at a time characterised by what has been termed a change in the biological NDD paradigm [7].

Hence, the purpose of this study was to conduct a comparative overview of age-specific incidence of NDDs, defined by their protein signature, in order to identify potentially systematic epidemiological patterns.

## Methods

### Disorders under study

We selected conditions constituting examples of NDDs in which pathognomonic protein deposits had been identified [8,9] (see Table 1 for a brief biochemical and epidemiological outline). In contrast, NDDs with an unclear biochemical signature, e.g., essential tremor, were excluded, despite the fact that incidence surveys were available [10,11]. In order to search for reported incidence measurements, we focused on: 1) sCJD, variant CJD (vCJD), accidentally transmitted CJD (atCJD), sRPNDd, ALS, FTD, PD, LBD and AD, as the most representative entities of the sporadic CNDD (sCNDD) group, 2) two genetic CNDDs (gCNNDs), namely, Huntington's disease (HD) and genetic transmissible spongiform encephalopathies (gCJD); and, 3) late, age-related, macular degeneration (AMD), a highly prevalent disorder presenting with amyloid deposits in drusen [9,12,13].

**Table 1:** Outline of epidemiological and biochemical features of the conformational neurodegenerative entities proposed for drawing up the document. Data obtained in part from reference [14] (a 1/10 genetic vs. sporadic incidence ratio is assumed).

Entity	Main protein deposit	Reported outbreaks		Annual incidence per million person-years		M / F	Evidence of transmission
				Sporadic	Genetic		
Creutzfeldt-Jakob	PrP^Sc^	vCJD	UK, Ireland, France, Spain	1	0.1	1.1 / 1	Yes
Amyotrophic lateral sclerosis and frontal lobe dementia	Ubiquitin, MAPT, SOD1, TDP-43 / FUS	ALS	Skaraborg county (Sweden) [15]. USA hGH-treated cohort [16].	10	1	1.5–2 / 1	No
Parkinson's disease and Lewy body disease	α synuclein	-	-	100	10	1.5–2.5 / 1	Yes^a^
Alzheimer's disease	β amyloid, Tau	-	-	1000	100	0.92 / 1	
Huntington's disease	Polyglutamine-rich huntingtin	-	-	~ 0	1	1	No
Late, age-related macular degeneration	EFEMP1 wild-type [12]		-	1000	?	0.95 / 1	No

^a^ Cell-to-cell transmission, from patient with Parkinson's disease to foetal cell graft.

### Incidence data sources

#### Literature review

**Fig 1:**
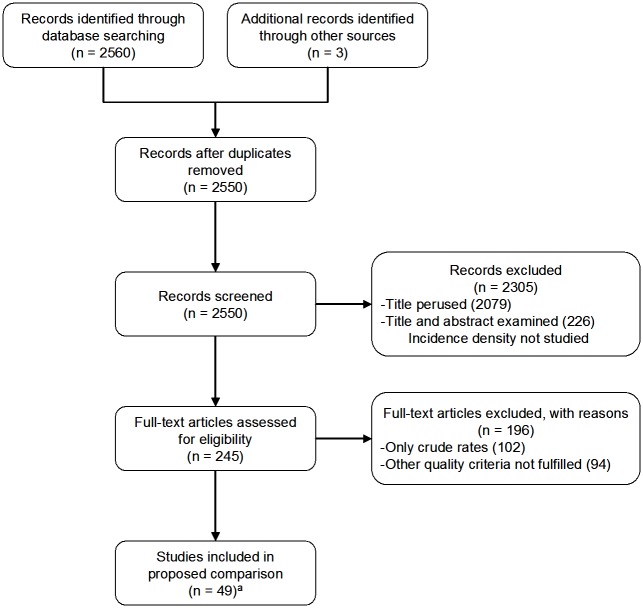
Flow chart showing search and selection of reports on incidence studies of eight conformational neurodegenerative disorders (including AMD). (^a^) A report on HD, which did not fulfil the quality criteria, was subsequently included.

**Fig 2:**
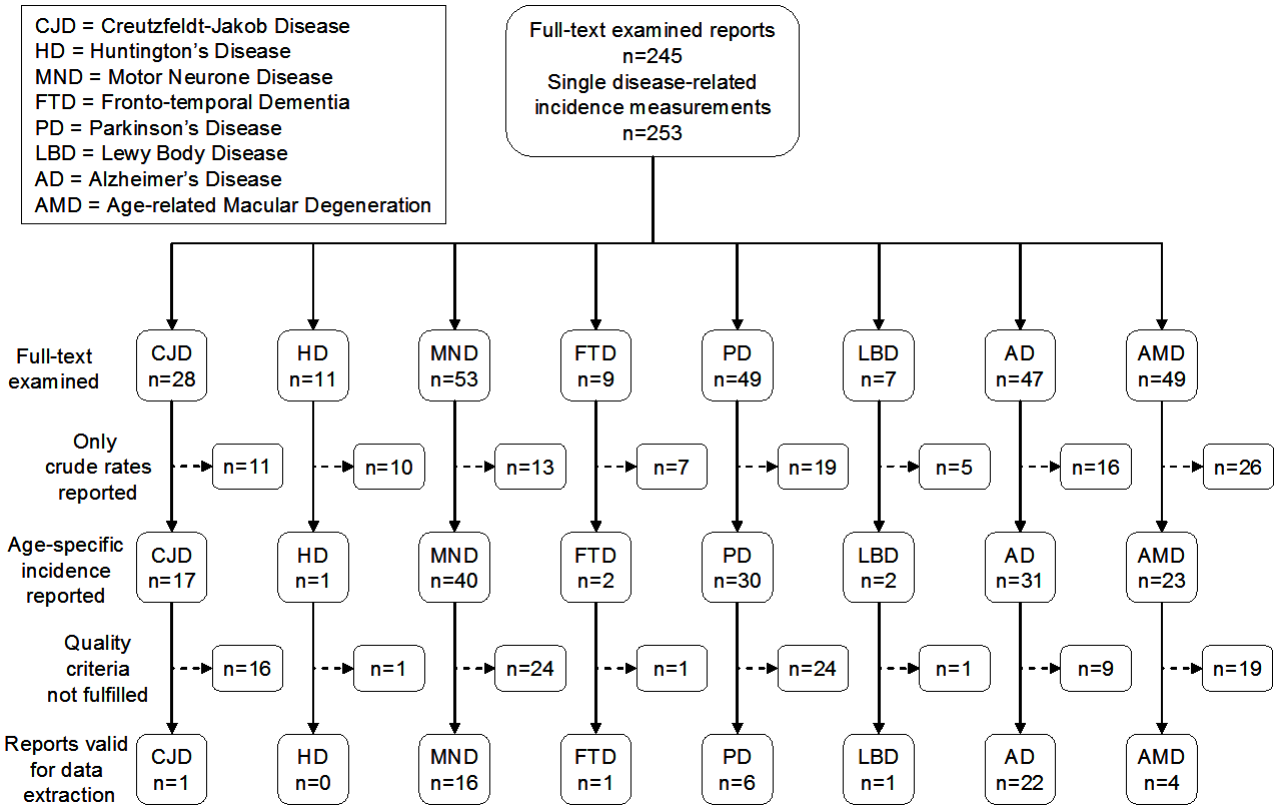
Flow chart showing selection of comparable incidence studies of conformational neurodegenerative disorders identified from the records reviewed.

For data-selection purposes, we identified research reports in MEDLINE from 1995 onwards, in all languages, using each of the following diagnostic search terms combined with *incidence*, i.e., *dementia*, *Creutzfeldt-Jakob syndrome*, *motor neurone disease (MND)*, *amyotrophic lateral sclerosis*, *fronto temporal dementia*, *Parkinson's disease*, *Lewy body disease*, *Alzheimer's disease*, *rapid progressive dementia*, *age-related macular degeneration* and *Huntington's disease*. Secondly, we examined titles and abstracts, searching for the presence of population-based incidence measurements. A flow chart is shown in Fig 1. Of a total of 2563 documents identified (three of which, all reported, were found among our own records), 2550 were not duplicated, and 2079 were discarded by consensus (JAI and JPC), on the basis of title content indicating a purpose other than general descriptive epidemiology. After examining 471 abstracts, 226 were rejected because they did not focus on incidence density, i.e., the ratio of new cases to the person-time of clinically unaffected individuals during a specific study period. Full-text examination yielded 253 incidence data sets for single disorders (Fig 2). Forty-nine reports met the following quality criteria for incidence measurements: 1) conditions under study either fulfilled explicit diagnostic criteria or the majority of diagnoses had been generated in national health systems with good access to neurological diagnosis, surveillance registries or figures obtained from models; 2) age-specific figures were available for 5-year age-groups; 3) screening methods were used in late-life NDD studies; and, 4) for screening surveys requiring pooled data sets, groups of consecutive age-specific intervals were available. A strict application of quality criteria to records subjected to full-text examination yielded 51 valid data sets for seven disorders, namely: CJD, 1; MND/ALS, 16; FTD, 1; PD 6; LBD 1; AD 22; and AMD 4 (Fig 2).

#### Complementary, requested or combined information

When the outcome of the literature review was deemed not to qualify for inclusion (e.g., study size too small for stable age-specific counts, or reports focusing on mortality rather than incidence of CJD), the required age-specific incidence counts for 5-year age groups (cases and, sometimes, person-year denominators) were obtained upon request from three sources: firstly from authors reporting ALS incidence on the basis of National Registry data in Sweden [17,18]; secondly, from the Research and Surveillance Unit at the Edinburgh Unit (RG Will) for atCJD and vCJD in the UK, for the period 1993–2007; and thirdly, from the Spanish CJD Registry 1995–2013 for gCJD, sCJD and sRPNDd (Table 2). For the last of these, we identified 104 post-mortem examined non-CJD-cases which had been diagnosed during the period 1995–2013 in Spain and initially reported to the National CJD registry as suspected CJD. Seventy-six out of the 104 had a copy of the neuropathological record at the National Registry, either as a result of notification or obtained on request from the regional registries. These records were reviewed by one of the authors, AR, and considered sufficient for biochemical protein profile classification in 51 instances. The 76 cases were characterised as follows: neurodegenerative, 14; vascular, 20; mixed (neurodegenerative + vascular), 23; inflammatory or metabolic, 10; tumour, two; and unclassified, seven. Incidence of registered sRPNDd in Spain across the period 1995–2013 was calculated on the basis of 36 neurodegenerative or mixed sRPNDd cases with valid data on age at onset and survival.

**Table 2:** Cases and population denominators used to generate incidence counts from epidemiological surveillance (vCJD, atCJD, sRPNDd, gCJD, sCJD), Swedish National registries (ALS) or selected surveys (PD) [17–23].

Age group	United Kingdom 1993–2007			Spain 1995–2013				Sweden 1991–2005		Sweden 2008–2011		Selected PD screening surveys									
	vCJD	atCJD		gCJD	sCJD	sRPNDd		ALS		FTD^a^		Ferrara		ILSA		NEDICES		PAQUID		POOLED	
Years	n	n	PYT	n	n	n	PYT	n	PYT	n	PYT	n	PY	n	PY	n	PY	n	PY	n	PY
**0–9**	0	0	109,772																		
**10–14**	5	0	56,575		1																
**15–19**	29	0	55,303		0		151,492^b^														
**20–24**	37	4	56,885	1	1		52,661														
**25–29**	35	15	61,747	0	1		59,687														
**30–34**	28	12	66,621	1	2		61,911	26	9,265												
**35–39**	15	10	66,468	8	4		59,236	37	9,204	1	6,843	3	263,158							3	263,158
**40–44**	5	3	62,238	9	10		54,456	55	8,993	2	3,421	12	275,862							12	275,862
**45–49**	3	2	59,163	14	25		49,118	107	9,251	2	3,421	20	289,855							20	289,855
**50–54**	6	0	55,202	19	54	1	44,021	202	8,943	18	2,369	40	287,563							40	287,563
**55–59**	0	0	50,744	19	97	1	39,027	304	8,013	26	2,341	64	304,907							64	304,907
**60–64**	1	0	44,238	19	140	5	36,707	401	6,728	58	2,391	96	219,529							96	219,529
**65–69**	1	0	39,826	11	190	3	34,300	524	6,027	69	2,292			8	3,613	1	1,481	7	2,026	16	7,120
**70–74**	2	0	35,735	4	194	5	31,277	649	5,733	49	1,560			8	3,345	10	4,439	11	5,297	29	13,081
**75–79**	0	1	28,580	1	174	7	25,158	583	5,015	69	1,157			10	2,834	7	3,151	23	7,198	40	13,183
**80–84**	0	0	20,169	3	57	7	16,948	410	3,776	51	845			16	2,360	6	2,008	18	6,298	40	10,666
**≥85**	0	0	16,491	0	20	7	13,565	155	3,157	7^**a**^	1,268^**a**^					6	1,640	9	5,000	15	6,640

^**a**^Data on the ≥85 age stratum were also obtained from the reported graph, in two strata: 85–89 years, n = 6, PY = 547; 90–99 years, n = 1, PY = 721

^**b**^Data refer to the 0–19 age group.

PYT = Person years in thousands

PY = Person years, number

NEDICES. Neurological Disorders in Central Spain study

ILSA Italian Longitudinal Study on Ageing

PAQUID. Personnes Agées QUID

#### Selection of single studies for comparison

From among the studies that met the inclusion criteria, one, usually the largest, was selected to represent each disease/disorder for comparison purposes. For AMD surveys, measurements corresponded to small-sized studies or to cohort follow-up studies, where baseline and incidence counts referred to different subgroups or stages of disease development. Other statistics referred to incidence of blindness from AMD, and so estimated incidences of late AMD in the UK were considered the most appropriate for comparison purposes [24]. Figures for AD were obtained from models, i.e., from reported graphs for AD [20,24]. PD figures were obtained for a composite population made up of <65-year-olds in Sardinia, and from screening surveys of subjects aged ≥65 years in three Italian, Spanish and French studies (ILSA, NEDICES and PAQUID, see Table 2 for interpretation of acronyms) [19,21–23]. Registry-based data obtained from the UK for vCJD and atCJD, from Spain for gCJD and sCJD, and from Sweden for ALS were deemed to provide the best-quality incidence measurements. For FTD and LBD, the only available quality study was included for each [18,23]. Available information failed to fulfil the quality criteria for either HD or sRPNDd. In the case of HD, there was a survey in which incidence figures were reported for 10-year age groups in Taiwan [25], with data not having been obtained for 5-year age groups. The data from the HD survey and the relatively scarce material on sRPNDd were deemed useful for comparative study purposes, to represent a group of 58 studies.

A summary view of the surveys (methodology, study-population, -area and -period), combined incidence figures in composite populations, and unreported studies furnished ad hoc are shown in Tables 2 and 3. Single data sets for vCJD, atCJD, gCJD, scJD, sRPNDd, HD, ALS, FTD, PD, LBD, AD and AMD cases, as well as the base population selected for comparison, are shown in Table 3. In general, incidence figures were based on binary outcome definitions of NDD diagnoses, frequently divided into two levels of certainty (definite and probable for prion disorders; or probable and possible in other CNDDs, when access to post-mortem diagnosis was infrequent). All basic information needed to calculate incidence or survival can be found in the papers cited or in the tables in this paper.

**Table 3:** Features of surveys/studies selected to represent conformational neurodegenerative disorders for the purpose of a comparative incidence study.

Entity	Reference/source	Study population, no. of individuals (population or cohort at baseline)	Incidence study period (years)	Age-group interval (no. years)	Defined entity under study	Diagnostic criteria	Total no. of cases	Truncated incidence per million person-years	Age-adjusted incidence per million person-years (all age-groups)
vCJD	UK CJD surveillance	59050466	1993–2007	5	vCJD	EuroCJD	167	0.19	0.09
atCJD	UK CJD surveillance	59050466	1993–2007	5	hGH-CJD	EuroCJD	47	0.05	0.03
gCJD	Spanish CJD surveillance	42356941	1995–2011	5	gCJD	EuroCJD	111	0.15	0.17
sCJD	Spanish CJD surveillance	42356941	1995–2011	5	sCJD	EuroCJD	975	1.34	1.55
sRPNDd	Spanish CJD surveillance	42356941	1995–2011	5	sRPNDd	Not specific, neuropathology	36	-	0.06
Huntington's disease	Taiwan Chen et al. 2010 [25]	22625000	2000–2007	10	HD 333.4-ICD-9	Included in SAD	165	0.91	1.15
Amyotrophic lateral sclerosis	Swedish ALS registry. Fang et al. 2009 [17]	8846523	1991–2005	5	*ICD-9*; 355C, *ICD-10*; G12.2	*ICD-9*; 355C, *ICD-10*; G12.2	3481	26.23	28.55
Fronto-temporal dementia ≥ 35y	Swedish National Registry. Nilsson et al. 2014 [18]	6977082	2008–2011	5	FTD (all subgroups)	The Lund and Manchester Group, and revised	352	12.61	10.10
Parkinson's disease	Pooled selected data [19,21–23]			5	PD	See below	375		564.3
35-65y	Granieri et 1991 [19]	1640874	1967–1987	5	PD	≥2 of four cardinal signs (as reported)	235	6.82	-
≥65y (study 1)	Perez et al. 2010 [23]. PAQUID	3777	1988–1993	5	PD	UKPDSBB criteria	68	2633	-
≥65y (study 2)	Benito Leon et al. 2004 [22]. NEDICES	5160	1994-1995/ 1997–1998	5	PD	≥2 of four cardinal signs if untreated	30	2358	-
65-84y (study 3)	Baldereschi et al. 2000 [21]. ILSA	434	1992-1993/ 1995–1996	5	PD	≥2 of four cardinal signs if untreated	42	3456	-
Lewy body dementia	Perez et al. 2010 [23]. PAQUID	3777	1988–1993	5	LBD	McKeith diagnostic criteria	29	1123	140.9
Alzheimer's disease	Gao et al. 1998 [20], meta-analysis	Not specified	1991–1997	5	Dementia and AD	DSM III, IV, and NINCDS-ADRDA for AD	Not specified	Not specified	2,588
Age-related macular degeneration	Owen et al. 2012 [24], estimations.	6990196	2007–2009	5	Late AMD	International Classification System or Wisconsin Age-Related Maculopathy Grading System and fundus photography	56700 estimated	3400	1,372

NEDICES. Neurological Disorders in Central Spain Study; ILSA. Italian Longitudinal Study on Aging; PAQUID. Personnes Agées QUID; UKPDSBB. United Kingdom Parkinson's Disease Society Brain Bank

### Statistical analysis

The number of cases used to calculate age-adjusted incidences for the overall general population differed considerably, namely, 36 for sRPNDd, 167 for vCJD, 47 for atCJD, 93 for gCJD, 3481 for ALS, 352 for FTD, 375 for PD and 29 for LBD (Tables 2 and 3). Age-specific incidence was assumed to be nil where no cases were reported. Age-truncated and directly age-adjusted incidences were calculated using the European Standard Population (Table 3). Since incidence counts were sometimes obtained from models or estimated from country-wide prevalence data [20,24], and survey data referred to population censuses or were based on population samples (i.e., PAQUID [23]), confidence intervals were not calculated [26] and no systematic statistical inter-entity comparison, such as the age-standardised rates ratio, was proposed. Calculations were performed using Microsoft Excel 2010.

We explored a potential relationship between incidence and disease progression, by obtaining median clinical disease duration in years (from clinical onset or, in some instances, from first medical visit to death), whether from reports (for atCJD from human growth hormone (hGH) treatments, and also for vCJD, sCJD, gCJD, HD, ALS, FTD, PD, LBD, AD) [24,25,27–30] or from registry records for sRPNDd. Survival in AMD was calculated as median life expectancy for estimated incident AMD patients in a UK population and life expectancy reported by Owen et al as supplementary data [24]. For the purpose of identifying patterns, figures for normalised age-specific and age-adjusted incidence and median survival were plotted on the same graph and examined visually.

### Ethics Statement

The only personal data used in this study corresponded to CJD surveillance activities by the Spanish Registry and were shown in grouped format (gCJD, sCJD and sRPNDd), in line with the policy of the regularly updated public domain website, http://www.isciii.es/ISCIII/es/contenidos/fd-servicios-cientifico-tecnicos/fd-vigilancias-alertas/fd-enfermedades/Informe_febrero_2015.pdf. Consequently, neither informed consent (Public Health General Law. BOE 2011, 240, Article 41.2 page 194613) nor ethical approval was required from the Carlos III Institute of Health Ethics Committee (*Comité de ética de la investigación y del bienestar animal*). Written consent for information to be stored at the Spanish Registry was not requested from patients or relatives, since notification is compulsory (National Epidemiological Surveillance Network Regulation governing human transmissible spongiform encephalopathies. *Orden 4093*, *BOE* 2001, 52, pp. 7676–7677), and JPC (CJD surveillance co-ordinator) and JAI have legally authorised access to individual registry data. Patient information was anonymised prior to statistical analysis. AR examined individual, identity-protected pathology reports supplied by the CJD Registry. Neither patients nor relatives were contacted. All CJD patients had died by the date of data-collection. No biological or tissue data were studied. The Spanish sRPNDd data set, grouped by age at onset, sex, diagnostic category and person-time denominators used for sRPNDd and for CJD, is available as a supplementary table (S1 Table).

## Results

### Age-adjusted and age-specific incidence

**Fig 3:**
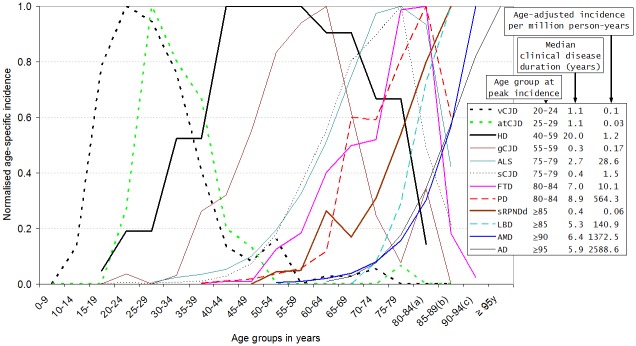
Normalised age-specific incidence, age-adjusted incidence and median clinical disease duration of Huntington's disease and genetic CJD as well as different sCNDDs, obtained either from reported data (ALS personally modified by Fang F) or from registries (the latter for atCJD mainly from treatments with human growth hormone), vCJD in the UK for 1993–2007 provided by RG Will, and registered gCJD in Spain for 1995–2011 obtained from J. de Pedro-Cuesta (not-reported). Other labelled data were obtained from reports [17,19–22,24,25,27–31]. Survival data sources: vCJD [27]; atCJD [28]; HD [30]; ALS, FTD, PD, AD and LBD [29]; AMD [24]. (a) 80–84 years is equivalent to 80 years and over for HD. (b) 85–89 years is equivalent to 85 years and over for sCJD, ALS, LBD, PD and sRPNDd. (c) 90–94 years is equivalent to 90 years and over for AMD and FTD. (d) 95–99 years is equivalent to 95 years and over for AD. Abbreviations: Creutzfeldt-Jakob disease (CJD); accidentally transmitted CJD (atCJD), genetic CJD (gCJD), Huntington's disease (HD), amyotrophic lateral sclerosis (ALS), motor neurone disease (MND), fronto-temporal dementia (FTD), Parkinson's disease (PD), Lewy body disease (LBD), age-related macular degeneration (AMD), Alzheimer's disease (AD), sporadic rapid-progressive neurodegenerative dementia (sRPNDd), sporadic conformational neurodegenerative disorders (sCNDDs).

As seen in Fig 3, there was a wide variation in age-adjusted incidence figures for sCNDDs, ranging from 0.03 per million person-years for atCJD to 2589 per million person-years for AD. With regard to the age-specific incidence curves, depicted as normalised incidences in Fig 3, two patterns were suggested: (a) inverted V-shaped curves, symmetrical or non-symmetrical, for entities with peak incidence at ages 80–85 years or lower, with few cases or modestly decreasing rates at the right-hand tail for FTD and PD respectively; or alternatively, (b) rising curves, systematically increasing with age for LBD, AD, and AMD. These patterns differed from the profiles seen: for HD, which affected all age-groups ≥15 years with a clear plateau; and for sRPNDd, which first appeared to parallel the rising curves of FTD or PD and then went on to mimic the profile of the age-related AD curve.

### Survival and age-specific incidence

Survival or clinical disease duration (Fig 3) varied almost 40-fold from median values, ranging from approximately 0.4–1.1 years for gCJD, sCJD, sRPNDd, vCJD and atCJD to a median of 20 years (95%CI 18.3–21.7) for HD [30], with intermediate values of 7.0 to 8.9 years for FTD and PD. In the case of acquired NDDs and sCNDDs other than sRPNDd, a direct relationship was suggested between age-adjusted incidence, survival and age at peak age-specific incidence. Incidence and survival were lowest for entities such as vCJD, atCJD and sCJD, whose incidence peaked at ages below 80 years, and ranged from 0.03 to 1.47 per million and 0.42 to 1.1 years respectively. Highest incidence and survival were seen for entities whose incidence peaked at ages above 80 years, particularly PD, LBD, AD and AMD.

### Protein deposits and age at incidence

The biochemical signatures of acquired CNDDs and sCNDDs (Table 1 and Fig 3) showed the following patterns: (a) lowest ages at peak incidences for PrP-related ailments, ranging from 20–24 to 75–79 years for vCJD, atCJD and sCJD; (b) a medium age-at-onset interval peaking from 75 to 85 years for *MAPT*, *FUSS*, *SOD 1* (frequent in ALS and FTD); and, (c) highest age at peak incidence for pathologies associated with α-synuclein, mixed α-synuclein+tau and Aβ+tau. For sRPNDd, no clear biochemical signature was suggested by age at onset (S1 Table) or survival (data not shown). HD, a biochemically distinct entity, with an incidence similar to sCJD, registered the longest disease duration by far. To summarise, it would appear that similar or shared protein signatures correspond to NDD forms with adjacent age-specific incidence curves, i.e., similar ages at onset, with a large variation for PrP, and heterogeneous, discordant patterns for proteins in the two genetic forms (gCJD and HD).

## Discussion

### Overall incidence variation

This study shows a more than 1000-fold variation in incidence of CNDDs. Results suggest that, concordant with survival and magnitude, there is an age-specific, inverted V-shaped profile shared by sCNDDs among the young and middle-aged, and an exponential-like increase for late-life conditions, with a transitional shape for FTD and PD. Distinct, discordant patterns are seen for HD and sRPNDd.

It must be assumed that, in all likelihood, incidence of sRPNDd will be a considerable underestimate of that of the wider sRPNDd spectrum [7]. Incidence counts for PD, LBD, AD, and perhaps even more so for ALS, in view of the recent identification of *C9ORF72* mutations in formerly sporadic cases [32], will constitute modest overestimates of the incidence of the corresponding sporadic form, given the low (5%-15%), albeit increasing, proportion of genetic forms frequently included in counts. This error, as well as the impact of the 2- to 3-fold variation in incidence within conditions (not reported here), might be attributable to increased disease awareness (e.g., in CJD), improvement in research methods (screening, registration), access to neurological diagnosis, and perhaps even improved diagnostic criteria. All things considered, including a potential causal role for local population- and entity-specific risk factors, we feel that such variation will not mask the approximately 10-fold sequential variation in incidence between biochemically different entities, i.e., sCJD, ALS, PD and AD. In sum, there is a large variation in incidence of CNDDs explained by incidence of the sporadic forms.

### Validity of incidence measurements

Viewed from the standpoint of neuropathological diagnosis, incidence measurements based on clinical grounds have limitations, e.g., considering the false-negative rates for Aβ and neurofibrillary degeneration among non-demented persons, as seen in the Nun Study [6], and the low predictive positive value for PD [4]. The complex fragmentary spectrum of late-life entities and the elderly brain, as seen from protein deposits reported by community studies [33], and the phenotypical convergence with disease progression [7], would suggest that taxonomic limits are sensitive to disease progression, thus undermining the permanent validity of clinically defined numerators. Since this pattern appears to be similar in part to that described for cognitively healthy elderly [6], and some of these deposits, i.e., tau, observed as early as the second decade of life [34], increase with age, the denominators of frequent late-life sCNDD incidence would not represent a disease-free population. Hence, the real incidence of biochemical abnormalities related to late-life NDDs can be assumed to be much higher and less specific than our clinically observed figures: this is particularly so at advanced ages, as a result of the considerable proportion of subclinically affected individuals at time of death, who, if clinically incident, should have been moved to numerators. This would also suggest that: (a) in mid-life, age-specific incidence of biochemically abnormal deposits is several-fold higher than that of corresponding clinical disease at the same age; and, (b) the time point of action of sCNDD initiators is during the early decades. By definition, clinical convergence makes the validity of incidence measurements temporary, in transition from what marks clinical or neuropathological lesion thresholds to always more complex cumulative phenotypes that are best observed with disease progression. For instance, after a several years of follow-up, a clinically prevalent PD case converted to neuropathologically-proven LBD [35].

### Age-specific incidence profile

**Fig 4:**
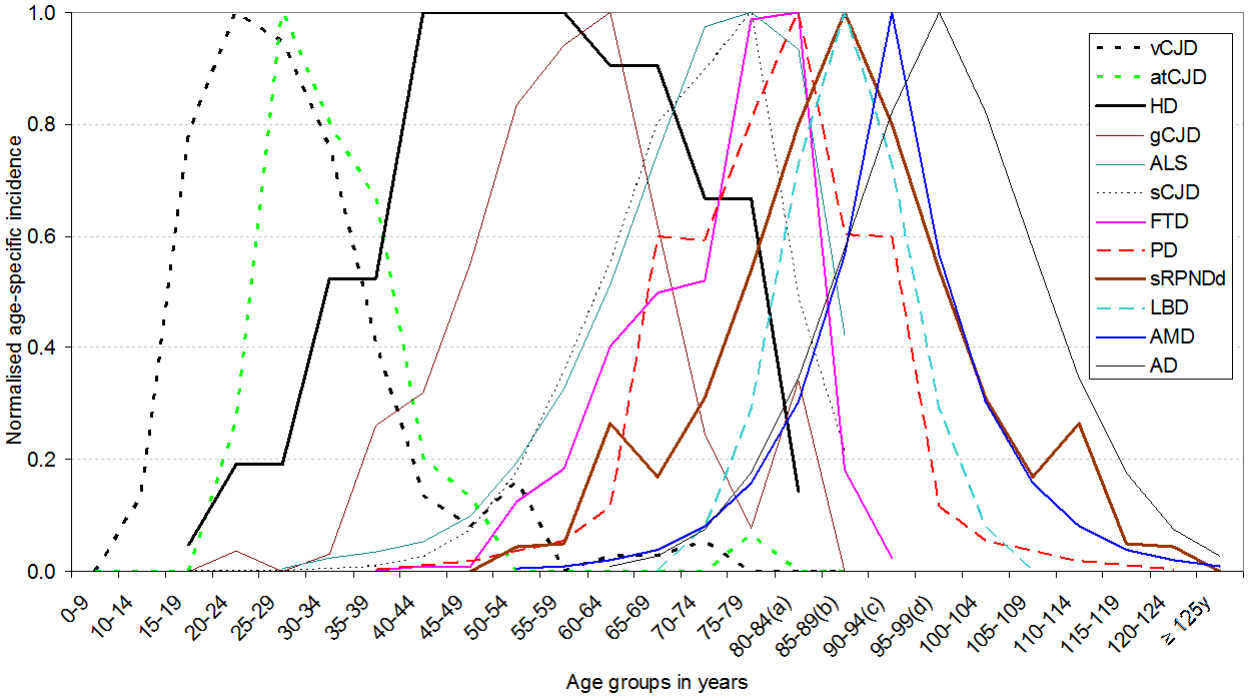
Reported and hypothesised (symmetrically replicated, dotted line) normalised incidence of sCJD, ALS, PD, LBD, AD, sRPNDd and AMD, assuming protracted population survival until 125 years for a substantial proportion of elderly. (a) 80–84 years is equivalent to 80 years and over for HD. (b) 85–89 years is equivalent to 85 years and over for sCJD, ALS. Abbreviations: Creutzfeldt-Jakob disease (CJD); accidentally transmitted CJD (atCJD), amyotrophic lateral sclerosis (ALS), motor neurone disease (MND), fronto-temporal dementia (FTD), Parkinson's disease (PD), Lewy body disease (LBD), age-related macular degeneration (AMD), Alzheimer's disease (AD), sporadic rapid-progressive neurodegenerative dementia (sRPNDd).

The traditionally assumed, direct relationship between NDD incidence and age was questioned early by Italian scientists in the case of ALS, and is readily observable in reports on all CJD forms. Not only is this reflected for ALS and FTD in our selected, large-scale Swedish studies [17,18], but it is also present in PD when registered diagnoses from medical services are studied [19,36] and in our pooled studies in which screening was used [19,22,23]. Evidence of AD and AMD incidence increasing with age is consistent with a large number of reports but has to be reconciled with: the slowing of dementia incidence after the age of 90 years, reported by seven of the eight studies recently reviewed by Kravitz et al. [37]; the prevalence at death of Braak stage, and microvascular brain lesions increasing with age in cognitively clinically silent individuals [6]; and the prevalence of pathological hallmarks of AD levelling off or even decreasing after the ninth decade of life (see Nelson P et al. [38] for a review). In a few quality surveys, we found both an increase in AD incidence with age in both sexes, and discordant AD incidence among persons aged 90 years and over, decreasing among men, and increasing and more stable among women [22,39,40]. The length of the human life span might be insufficient for the purpose of fully observing sCNDD incidence among the oldest old. Interpretation of the incidence profile with advanced age seen for late-onset sCNDDs has to be considered on the basis of post-mortem data. In accordance with the increasing prevalence at death occurring at ages >85 years observed for argirophylic grains (100% among centenarians), for primary age-related tauopathy and for our incomplete sRPNDd counts, we propose a graphical interpretation of the general age-at-onset-related pattern of sCNDDs, based on a simulation (Fig 4) of longer life-expectancy with a frequent survival until 125 years (dots), by adding symmetrical age-specific values for PD, LBD, AD and AMD to suggest that clinically silent post-mortem findings converted to incident clinical cases [17,19–22,31]. In brief, we propose that acquired, sporadic and some genetic CNDDs display similar incidence features with fairly constant subclinical-to-clinical conversion rates, until the age at which the highest proportion of individuals reach the threshold for clinical signs. Subclinical lesions wearing off at ages over 80 years would occur for clinically fulminant entities, such as sCJD and ALS.

There is a tradition of statistical testing in assessment of changing trends in late-life sCNDD incidences. We believe that, given the uncertain validity of such counts, a merely statistical discussion of age-specific profiles is to be discouraged. Similarly, statistical testing of the fitness of age-specific data for different functions, e.g., log-normal or Gaussian curves for acquired CJD, and exponential or sigmoid functions for sCNDDs incident in late life warrant a detailed, monographic approach. Additionally, the considerable age-specific heterogeneity of an inter-entity comparative incidence measure, particularly when applied to acquired CJD forms and late-life sCNDDs, questions the wisdom of using a single summary measure. It would naturally obscure valuable information of our proposed age-at-onset, changing, systematic pattern for sCNDD incidence, and survival would remain disregarded.

### Phenotypical convergence

Phenotypical convergence, as a major element of the paradigm distinguished by Warren et al. [7], might in part have been perceived as a result of refinements in clinical methods. For instance, clinicians have recently become aware of: (a) the frequency of non-motor symptoms in entities traditionally defined by movement or motor-function impairments, such as PD and ALS [41,42]; and, (b) the presence of motor symptoms in traditionally cognitive sCNDDs [43–45]. Furthermore, clinical, morphological and biomarker research has threatened the notion of taxonomic limits existing between PD, PD with dementia and LBD, and between ALS and FTD [46,47]. Here, we avoid a traditional discussion on the incidence of vascular or mixed vascular-AD dementia, referring the reader to the wider phenomenon of frontiers of organ-specific amyloid disorders. Similar misfolded protein deposits in nervous system and vascular structures, i.e., Aβ in AD [48,49] and PrP in sCJD [50], challenge the concept of such entities as organ-limited amyloid disorders. The excess vascular risk of AD might be attributable to its angiopathy becoming symptomatic prior to the clinical expression of CNS lesions [51].

### Conclusions

The strength of our study lies in its wide coverage of entities. Among its weaknesses, it should be noted that the study is at times supported by insufficient data. Bias must inevitably stem from selecting a small set of studies to address the wide panorama of CNDDs.

To sum up, our results support the notion that the molecular nexopathy paradigm has implications for interpreting NDD epidemiology [3]. Accordingly, the age-specific incidence of sCNDDs in late-life might reflect the presence of several, interrelated, conformational disorders representing fragments of a similar overlapping endpoint. The biochemical-signature-related pattern appears to provide more elements for a unified than for a fragmentary view of the acquired and sporadic CNDDs forms. The strong pathophysiologically-oriented nature of the molecular nexopathy paradigm as originally formulated [7] could be completed with the epidemiological view, thereby providing a more unified panorama for prevention. If so, a similar cross-entity approach to the analytical epidemiology of sCNDDs might be fruitful.

## Supporting Information

S1 Table Age at clinical onset and neuropathology of 76 CJD excluded notifications with available post-mortem study report. (DOC)
