# Correction: Comparative Incidence of Conformational, Neurodegenerative Disorders

**DOI:** 10.1371/journal.pone.0140304

**Published:** 2015-10-06

**Authors:** Jesús de Pedro-Cuesta, Alberto Rábano, Pablo Martínez-Martín, María Ruiz-Tovar, Enrique Alcalde-Cabero, Javier Almazán-Isla, Fuencisla Avellanal, Miguel Calero

The image for Fig 4 is incorrect. Please see the corrected Fig 4 here.

**Fig 4:**
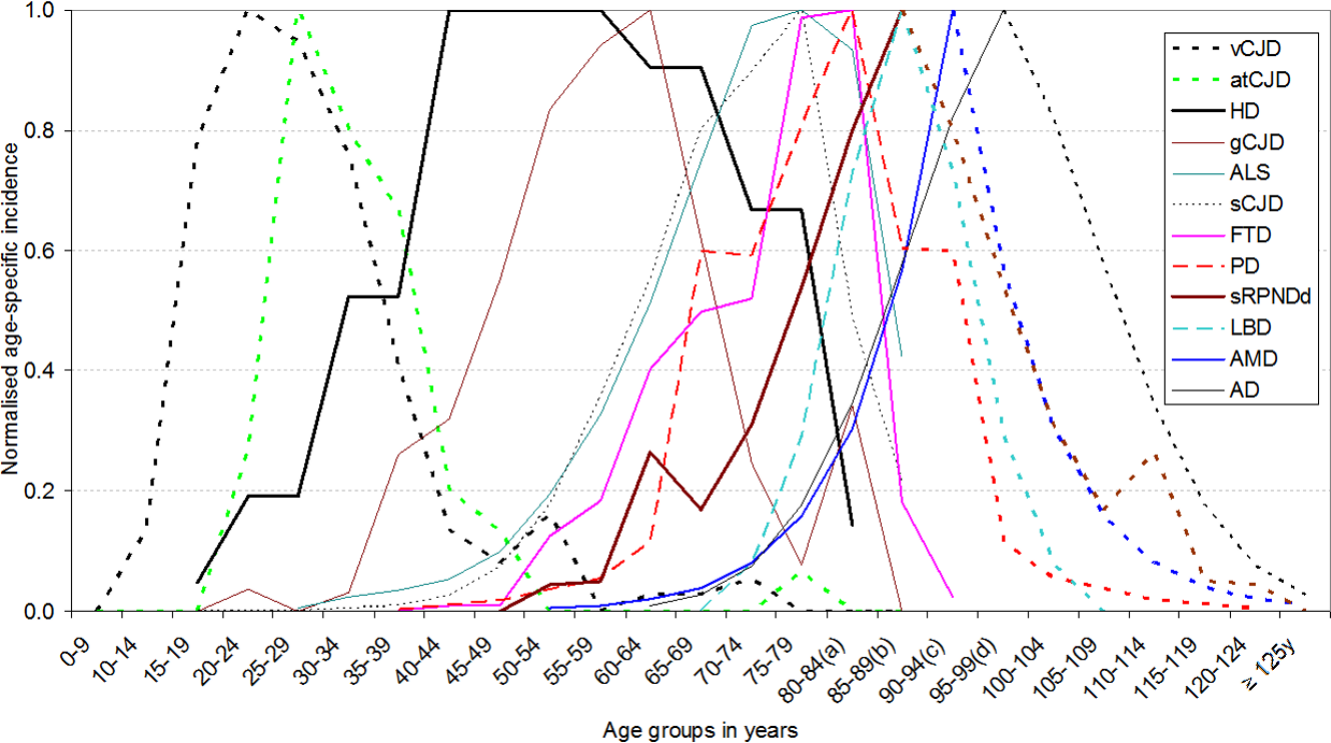
Reported and hypothesised (symmetrically replicated, dotted line) normalised incidence of sCJD, ALS, PD, LBD, AD, sRPNDd and AMD, assuming protracted population survival until 125 years for a substantial proportion of elderly. (a) 80–84 years is equivalent to 80 years and over for HD. (b) 85–89 years is equivalent to 85 years and over for sCJD, ALS. Abbreviations: Creutzfeldt-Jakob disease (CJD); accidentally transmitted CJD (atCJD), amyotrophic lateral sclerosis (ALS), motor neurone disease (MND), fronto-temporal dementia (FTD), Parkinson's disease (PD), Lewy body disease (LBD), age-related macular degeneration (AMD), Alzheimer's disease (AD), sporadic rapid-progressive neurodegenerative dementia (sRPNDd).
