# Supplementary material for: Comparative Incidence of Conformational, Neurodegenerative Disorders
Source: PLoS One. 2015 Sep 3;10(9):e0137342. doi: 10.1371/journal.pone.0137342 (PMC4559310; doi:10.1371/journal.pone.0137342)
Supplement: S1 Table — (DOC) [file pone.0137342.s001.doc]

S1 Table. Age at clinical onset and neuropathology of 76 CJD excluded notifications with available post-mortem study report.

Age at onset and biochemical profile of 36 of these classified as incident, sporadic, rapidly progressing neurodegenerative (or mixed) dementia.

|  |  | Age at onset in years – number of patients | | | | | | | | | |
| --- | --- | --- | --- | --- | --- | --- | --- | --- | --- | --- | --- |
|  |  | 0-49 | 50-54 | 55-59 | 60-64 | 65-69 | 70-74 | 75-79 | 80-84 | 85> | All ages |
| Neuropathology pattern | Neurodegenerative (ND) | - | 1 | 1 | 1 | 1 | 2 | 4 | 3 | 1 | 14 |
|  | Vascular | - | 1 | - | 1 | 7 | 7 | 1 | 3 | - | 20 |
|  | Mixed (ND+vascular) | - | - | 1 | 4 | 2 | 3 | 3 | 4 | 6 | 23 |
|  | Other (*) | 1 | 2 | 3 | 2 | 4 | 3 | 1 | 3 | - | 19 |
|  | All | 1 | 4 | 5 | 8 | 14 | 15 | 9 | 13 | 7 | 76 |
|  |  |  |  |  |  |  |  |  |  |  |  |
| Main deposit | α-synuclein | - | - | 1 | - | 1 | 3 | 4 | 3 | 2 | 14 |
|  | Tau | - | 1 | - | 1 | - | - | 1 | 2 | 1 | 6 |
|  | TDP-43 | - | - | - | - | - | - | - | 1 | - |  |
|  | Mixed or not studied | - | - | - | 4 | 2 | 2 | 2 | 1 | 4 | 15 |
|  | All | - | 1 | 1 | 5 | 3 | 5 | 7 | 7 | 7 | 36** |

*Inflammatory, metabolic, neoplasms, and seven unclassified.

** One neurodegenerative or mixed case was dropped due to incomplete information on year of onset of clinical symptoms
